# Biochar application significantly affects the N pool and microbial community structure in purple and paddy soils

**DOI:** 10.7717/peerj.7576

**Published:** 2019-09-13

**Authors:** Shen Yan, Zhengyang Niu, Haitao Yan, Fei Yun, Guixin Peng, Yongfeng Yang, Guoshun Liu

**Affiliations:** 1 Department of Tobacco cultivation, Henan Agricultural University, Zhengzhou, Henan, China; 2 Henan Biochar Engineering Technology Research Center, Zhengzhou, Henan, China; 3 Henan Biochar Technology Engineering Laboratory, Zhengzhou, Henan, China; 4 Department of Microbiology, Cornell University, Ithaca, NY, USA; 5 China Tobacco Henan Industrial Co.,Ltd., Zhengzhou, Henan, China

**Keywords:** Biochar, Microbial community, Paddy soil, Nitrogen cycle, Purple soil

## Abstract

**Background:**

The increasing demand for food production has resulted in the use of large quantities of chemical fertilizers. This has created major environmental problems, such as increased ammonia volatilization, N_2_O emission, and nitrogen (N) leaching from agricultural soil. In particular, the utilization rate of N fertilizer is low in subtropical southern parts of China due to high rainfall. This causes not only large financial losses in agriculture, but also serious environmental pollution.

**Methods:**

In this study, 16S rDNA-based analysis and static-chamber gas chromatography were used to elucidate the effects of continuous straw biochar application on the N pool and bacteria environment in two typical soil types, purple and paddy soils, in southern China.

**Results:**

Straw biochar application (1) improved the soil N pool in both rhizosphere and non-rhizosphere soils; (2) significantly reduced the emission of N_2_O, with no difference in emission between 1 and 2 years of application; (3) increased the abundance of N-processing bacteria in the soil and altered the bacterial community structure; and (4) improved the tobacco yield and N use efficiency in paddy soil. These findings suggest that, in southern China, the application of straw biochar can promote N transformation in purple and paddy soils and reduce the emission of the greenhouse gas N_2_O.

## Introduction

Nitrogen (N) is an essential element for plant growth and an important agricultural input in soil. To maximize food production, farmers often apply N-fertilizer at rates higher than those required by plants (Chen et al., 2018; Mavi et al., 2018). This practice is common in the subtropical regions of South China, where high rainfall and soil leaching ability lead to the low utilization rate of N fertilizers, causing not only substantial losses to the agricultural industry, but also serious environmental pollution, such as acidification of soil, eutrophication of surface water (Feng et al., 2018), and emission of the greenhouse gas N_2_O (Dong et al., 2018; Gillette et al., 2018; Hou, Li & Zhao, 2009). Studies have reported that the average application rate of N fertilizers in tobacco fields in southern China is 135 kg N ha^−1^ yr^−1^, which is considerably higher than that in North Carolina in United States (80 kg N ha^−1^yr^−1^), where the rainfall is less than that in southern China (Drake, Vann & Fisher, 2015; Zhu et al., 2018).

Biochar refers to a class of highly aromatic refractory solid materials produced by pyrolytic carbonization in the complete or partial absence of oxygen; biochar is typically alkaline, highly porous, has many functional groups, and a large specific surface area (Tan et al., 2016). Biochar can be added to soil as a soil conditioner. It can alter a wide variety of soil properties (Abujabhah et al., 2018; Anderson et al., 2011; Walters & White, 2018), including soil water-holding capacity, microbiology community, bulk density, pH, and soil C and N content, because of its porous structure, high surface area, and affinity for charged particles (Keech, Carcaillet & Nilsson, 2005). It also affects soil N cycling (Jien et al., 2018). First, direct adsorption of N to biochar in the soil reduces the matrix material available for nitrification and denitrification, and the adsorption of microbial inhibitors can catalyze various biochemical reactions (Anderson et al., 2011; Clough & Condron, 2010; Kammann et al., 2017). Second, several elements and trace elements in biochar affect the community structure, functional diversity, and N cycling microorganism activities. Biochar helps improve soil N fixation and reduces greenhouse gas emission by promoting biological N fixation in the soil and inhibiting denitrification (Anderson et al., 2011; Ball et al., 2010; Cusack, Silver & McDowell, 2009; Prommer et al., 2014). Third, biochar releases volatile organic compounds in soil, thereby affecting soil ammoxidation and nitrous oxide oxidation (Dempster et al., 2012; Humbert, Zopfi & Tarnawski, 2012; Prommer et al., 2014; Zhu et al., 2011). Finally, biochar alters the physical and chemical properties of soil, by changing pH and porosity; this affects microbial habitats and can provide a favorable environment for the growth and reproduction of aerobic bacteria such as nitrifying and N-fixing bacteria, thereby promoting the N cycle (Singh et al., 2010; Yanai, Toyota & Okazaki, 2007; Zhang et al., 2010).

Moreover, biochar can promote crop growth (Asai et al., 2009; Chan et al., 2008). It can interact with the soil and can have cascading effects throughout the ecosystem (Hammes & Schmidt, 2009), which can affect plant growth significantly. Furthermore, biochar can stimulate crop root growth (Prendergast-Miller, Duvall & Sohi, 2014), improve C uptake through photosynthesis (Xu et al., 2015), and increase crop production (Asai et al., 2009). Biochar is a byproduct of bioenergy production. It can increase crop production and reduce fertilizer use. Biochar is considered an ideal solution to global environmental challenges (Laird, 2008). However, there are large differences in how plants and soils respond to biochar, and it is not clear how plants respond to the application of biochar (Biederman & Harpole, 2013). Paddy and purple soils are the most important soil types for crop production in southern China, including in the main tobacco planting areas. These two kinds of soil are negatively affected by N fertilizer application for tobacco planting in southern China. Thus, the objectives of this study were to determine the effects of straw biochar on the soil N pool and N-processing microbial community in tobacco fields in southern China using a field experiment. We hypothesized that (a) the effect of biochar application on soil alters the N pool and changes the community of nitrifying and denitrifying microbes, and (b) biochar application promotes the N utilization rate and improves tobacco production.

## Methods

### Test site

The test site is located in a tobacco production field, in Xinfeng, Jiangxi Province (paddy soil: N:25°27′11.71″, E:114°51′54.25″purple soil: N:25°26′48.33″, E:114°51′28.82″). The climate in the area is subtropical moist monsoon. The average annual number of sunshine hours was 1,473.3–2,077.5 h, with an average annual temperature of 18 °C–19.7 °C, a frost-free period of 250 d, and an average annual rainfall of 1,410–1,762 mm. The paddy and purple soils are classified as Typical Stagnic Anthrosols and Pup-Orthic-Entisol, respectively, in the Chinese Soil Taxonomy (Gong, 1999). Data of the nutrient status in the test site are presented in Table 1.

**Table 1:** Nutrient status of the experimental soils for paddy soil and purple soil.

Soil type	Organic matter (mg g^−1^)	Hydro-N (mg kg^−1^)	Available P (mg kg^−1^)	Available K (mg kg^−1^)	pH	Total C (mg g^−1^)	Total N (mg g^−1^)
Paddy soil	27.04	129.80	24.20	109.51	5.59	16.60	2.10
Purple soil	9.67	43.31	7.09	223.54	7.30	15.00	0.70

### Experimental design

The experiment was carried out in early March 2017. Biochar was applied to the soil surface at a rate of 7.2 t ha^−1^, and mixed with the topsoil (0–20 cm). In treatment 1 (T1), the application was left for 1 year (2016). In treatment 2 (T2), it was left for 2 years (2016 and 2017). As a control (T0), soil was left untreated (that is, with no biochar) for two years. The amount of biochar applied was based on the amount used in tobacco fields in previous years, which resulted in the best tobacco yield (Table S1). Each experimental field covered an area of 666 m^2^. The trial was performed in triplicate in a random block arrangement, with an extra block without N application, to measure N use efficiency (NUE). Yunyan 87 tobacco variety plants were transplanted in early March (Table S2) at a spacing of 50 cm ×120 cm. The accepted local high-quality tobacco production technology was applied (Xiao et al., 2003). Furthermore, 135 kg ha^−1^ of pure N (N:P:K = 1.0:0.9:2.8) was applied to the paddy soils and 142.5 kg ha^−1^ of pure N fertilizer (N:P:K = 1.0:1.0:2.8) to the purple soils. The biochar (Table S3) was provided by Sanli New Energy Co. Ltd. (Shangqiu, China); it was prepared from rice straw subjected to 350 °C heating for 30 min under anoxic conditions in a continuous carbonization furnace.

### Sample collection and analytical methods

#### Sample collection

Soil samples were collected 60 d after planting. Five representative strains of tobacco from each plot were pulled from the soil, including the roots and soil. The surface soil was shaken off the plants and the soil that adhered to the root surface was considered the rhizosphere soil (Fangdong, Yingang & Guojing, 2005; Smalla et al., 2001). Subsequently, the soil was washed from the tobacco roots, and the tobacco samples were dried in an oven to determine the dry weight (Voisard et al., 1989). The soil samples from the 0–20- and 20–40-cm soil layers were also obtained using a soil sampler. Approximately 100 g of fresh soil was refrigerated at 4 °C, passed through a 10-mesh sieve, and used to determine the amount of N in the soil derived from microbial biomass, ammonia, and nitrate. Fifteen samples of rhizosphere soil were mixed and the abundance of soil microbes was determined. Approximately 100 g of soil sample was dried under shade to determine other indicators.

#### Soil N analysis

The total soil N content was determined using a C/N elemental analyzer (Vario MAX, Elementar, Germany) and Sulfanilamide was used as quality control. Soil available N (AN) was measured according to the method of Lu (2000). The concentration of NH_4_^+^ and NO_3_^−^ was measured using a continuous-flow auto-analyzer (AutoAnalyser3; Seal Analytical, Norderstedt, Germany) and 100 mg/L (NH_4_)_2_SO_4_ and 200 mg/L KNO_3_ were used as quality control, respectively. Soil microbial biomass N (MBN) was estimated using the chloroform fumigation extraction method (Vance, Brookes & Jenkinson, 1987).

#### Metagenome analysis of soil microbial community structure

##### DNA extraction and PCR amplification.

Microbial DNA was extracted from 0.5 g of soil samples using the E.Z.N.A. Soil DNA Kit (Omega Bio-tek, Norcross, GA, USA) according to the manufacturer’s protocols. The V3-V4 region of the bacterial 16S ribosomal RNA gene was amplified by PCR. The PCRs were performed using the primers 341F (5′-CCTAYGGGRBGCASCAG-3′) and 806R (5′-GGACTACNNGGGTATCTAAT-3′). The amplification yielded an expected 16S V3-V4 region sequence length of about 465 bp.

##### Illumina MiSeq sequencing and analysis.

Amplicons were extracted from 2% agarose gels and purified using the AxyPrep DNA Gel Extraction Kit (Axygen Biosciences, Union City, CA, USA) according to the manufacturer’s instructions. The purified amplicons were pooled in equimolar quantities and paired-end sequenced (2 × 250 bp) on an Illumina platform according to the standard protocols (15035786 v01, https://www.illumina.com/). The operational taxonomic units were obtained from tags using cluster analysis, and a Ribosomal Database Project classifier was adopted for the accurate annotation of tags and operational taxonomic units.

#### Bioinformatics analysis

Raw data containing adapters or low-quality reads can affect the subsequent assembly and analyses. Paired-end clean reads were merged as raw tags using FLASH (v. 1.2.11) with a minimum overlap of 10 bp and mismatch error rate of 2% (Magoč & Salzberg, 2011). Noisy sequences of raw tags were filtered using QIIME 2 (Bolyen et al., 2018) pipeline under specific filtering conditions (Bokulich et al., 2013) to obtain high-quality clean tags. Reference-based chimera checking of clean tags was performed using UCHIME algorithm (http://www.drive5.com/usearch/manual/uchime_algo.html) in the reference database (http://drive5.com/uchime/uchime_download.html). The effective tags were clustered into OTUs of ≥ 97% similarity using UPARSE pipeline.

#### N_2_O emission measurement

N_2_O emission was measured by static-chamber gas chromatography. Gas samples were collected every 15 d from static chambers at check-points set on a ridge between two tobacco plants; three static chambers were placed per plot. The measurements were recorded eight times, from before tobacco transplant to harvest. The dimensions of the static chamber were in accordance with Dyer (2010): the chamber has a 20 cm diameter hole in its cover; the hold is plugged with a rubber stopper; there is an airway (six mm diameter, 10 cm length) near the hole that is used for balancing the pressure. The conduct operation of measuring emissions occurred between 9 am and 11 am in good weather, and three complete gas measurements were obtained at three time points: *t* = 0, *t* = 15, and *t* = 30 min. Before gas collection, a 50 ml injector was connected to the sampling window. Subsequently, the injector plunger was pushed three times to mix the gas in the static chamber. Finally, after the gas collection was completed, the injector was put inside a hermetic bag and brought back to the laboratory. Gas chromatograph analysis (TRACEGC 2000, Italy) was used to analyze the samples. N_2_O was detected using an electron capture detector (ECD); the test temperature was 300 °C, the boiler temperature was 80 °C, and the carrier was pure N_2_.

The accumulated emissions were calculated according to the following equation (Singh, Singh & Kashyap, 1999):

\begin{eqnarray*}\text{Accumulated}{\mathrm{N}}_{2}\mathrm{O}\text{emission}=\sum _{i}^{n}({F}_{i}\times {D}_{i}) \end{eqnarray*}

where *F*_i_ is the rate of N_2_O emissions flux (g m^−2^ d^−1^) in the *i*th sampling interval, *D*_i_ is the number of days in the *i*th sampling interval, and ‘*n*’ is the number of sampling intervals.

N_2_O emissions flux (g m^−2^ d^−1^) was calculated according to the following equation (Singh, Singh & Kashyap, 1999):

\begin{eqnarray*}F=(V/A)\times (\Delta c/\Delta t)\times (273/T) \end{eqnarray*}

where *V* is the volume of the chamber (m^3^), A is the area from which N_2_O emits into the chamber (m^2^), Δ*c*∕Δ*t* is the rate of N_2_O gas accumulation in the chamber (mg m^−3^ h^−1^), and *T* is the absolute temperature calculated as 273 plus the mean temperature in the chamber (°C).

### Statistical analysis

We used R v. 3.1.2 (R Core Team, 2013) to generate thermographs and Venn diagrams, and for one-way analysis of variance (ANOVA). Origin 9.0 was used to generate column plots.

**Figure 1:**
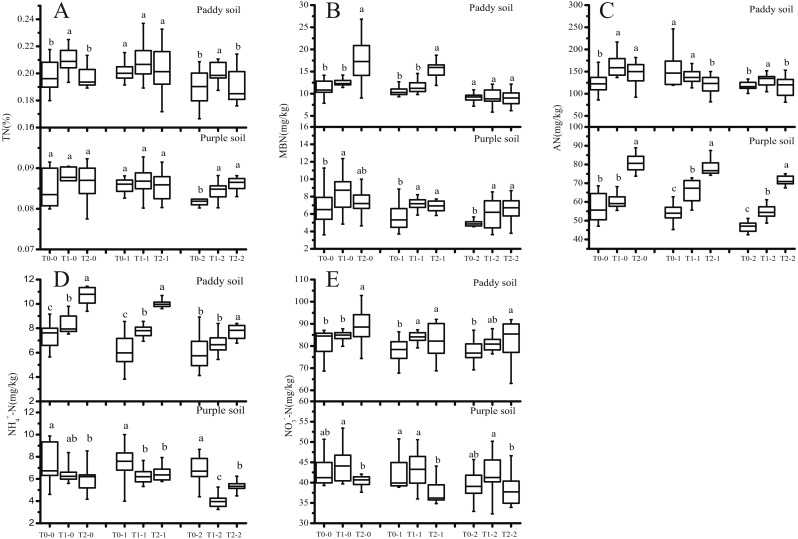
Effect of straw biochar on the N content in the paddy and purple soils of tobacco fields. T0, T1, and T2: control, 1-year, and 2-year biochar application, respectively; −0, −1, and −2: rhizosphere soil, 0–20, and 20–40 cm soil layers, respectively; (A) total N; (B) microbial biomass N; (C) available N; (D) N from NH${}_{4}^{+}$; (E) N from NO_3_. The letters above the bars indicate treatment groups, between which the values differed significantly at the 0.05 significance level (*n* = 15; LSD test); the lines in the box are the mean values.

## Results and discussion

### Effect of biochar on the N pool in the paddy and purple soils

Biochar affected soil N, and the effects differed between the paddy and purple soils. Biochar application had negligible effect on the content of nitrate-derived N, especially in the purple soil (Fig. 1A). However, biochar positively affected soil microbial biomass N in both soil types (Fig. 1B). Compared with that of T0, 2 years biochar application significantly increased the microbial biomass by 52.63% (*P* = 9.68E^−5^) in the rhizosphere soil and 53.22% (*P* = 2.03E^−8^) in the 0–20-cm soil layer in the paddy soil and 9.43% (*P* = 0.30) in the rhizosphere soil and 23.64% (*P* = 0.00071) in the 0–20-cm soil layer in the purple soil. This is because biochar application improved soil microbial biomass supply, especially organic matter, thus stimulating microbial activities to increase the biomass (Harter et al., 2014). Alternatively, it is possible that the higher C/N ratio results in higher N fixation activity of the microorganisms (Glaser, Lehmann & Zech, 2002). In addition, P, Cu, and Zn, which occur abundantly with biological C, also stimulate the growth of N fixing microorganisms (Keeling, Cook & Wilcox, 1998). Biochar increased the content of available N (Fig. 1C). Especially in the purple soil, the content of available N was improved with the duration of biochar application, and the content of available N improved by 40.74% (*P* = 1.44E − 11), 43.86% (*P* = 2.66E − 16), and 52.62% (*P* = 9.97E − 22) in the rhizosphere soil, 0–20-cm, and 20–40-cm soil layers, respectively. Biochar application improved the soil available N because its specific surface area and porous characteristics help retain N compounds in the soil. Furthermore, biochar application alters the physical and chemical properties of soil such as soil porosity and nutrition, thereby directly or indirectly affecting the diversity, abundance, and activity of microorganisms, and this influences the circulation and availability of soil N. Our finding that biochar application improved soil microbial biomass is consistent with the results of previous studies (Liang et al., 2010; Wardle, Nilsson & Zackrisson, 2008).

In the present study, biochar application affected ammonia-derived (Fig. 1D) and nitrate-derived (Fig. 1E) N levels differently in the paddy and purple soils; in the paddy soil, biochar significantly increased N from these sources, whereas the opposite was observed in the purple soil. This result can be attributed to the balancing effects of biochar on soil NH_3_ volatilization. On the one hand, the high alkalinity of biochar may help increase soil pH and alter soil aeration, thereby increasing NH_3_ volatilization (Sun et al., 2014). On the other hand, biochar can adsorb both NH_4_^+^ and NH_3_ because of its large surface area and because it contains various functional groups; this can help reduce NH_3_ volatilization (Mandal et al., 2016). Because of the large difference in pH between the two soil types, biochar application may affect NH_4_^+^ levels differently in these soils. In the paddy soil, the application of biochar makes the soil further acidic, which is unfavorable for NH_3_ volatilization. At the same time, the addition of biochar promotes the absorption of NH_4_^+^ and NH_3_ by the soil, which increases the content of ammonia-derived N. On the contrary, the purple soil is already alkaline, and the addition of biochar further promotes the increase in soil pH, increasing NH3 volatilization. In the paddy soil, the content of nitrate-derived N was significantly increased after the application of biochar, possibly because the soil moisture content in paddy soil is considerably higher than that in purple soil, and biochar application helps prevent the loss of N through leaching in the soil solution (Sun et al., 2018). However, in the purple soil, nitrate-derived N level was reduced after biochar application; this decline increased with the reduction in N fertilizer application, possibly because purple soil has small soil particles, which can lead to increased N loss by leaching when biochar is applied (Wenjuan, Yonghao & Jiyong, 2013).

In addition, in the present study, we found that although the soil N content decreased with increasing soil depth, the application of biochar promoted soil N pools in 0–20- and 20–40-cm soil layers. As the roots of tobacco are mainly concentrated in the 0–40-cm soil layer, the addition of biochar is beneficial to the absorption of N by tobacco, which is more conducive for the growth of tobacco. This result is consistent with the results of previous studies (Jiang-Zhou et al., 2015; Zhang et al., 2016).

**Figure 2:**
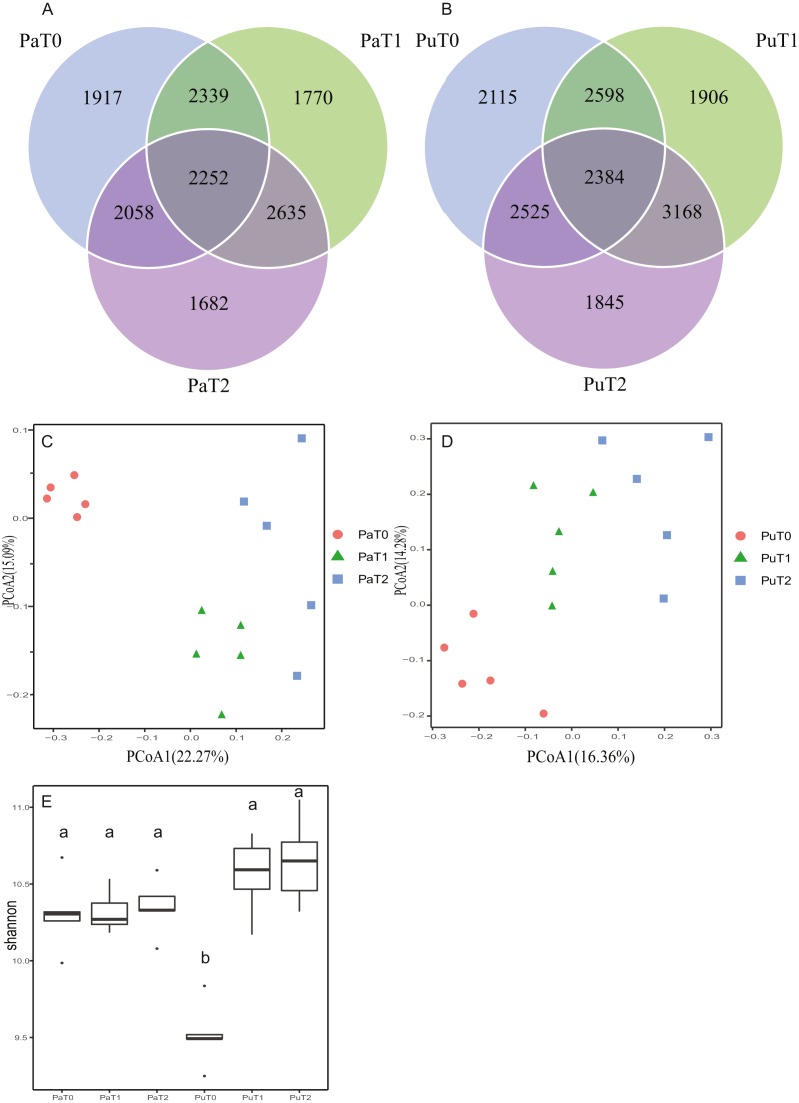
Biochar application effects on community structure and diversity of microbes in the paddy and purple soils. (A) and (B) Venn diagrams displaying the number of microbial operational taxonomic units shared between the control and the 1 and 2 year biochar application treatments. Principal coordinates analysis (C and D) and Shannon diversity indices (E) of different treatments in the soil microbiome. The letters above the bars identify groups of treatments between which the values differ significantly at the 0.05 significance level (LSD test).

### Effect of straw biochar on N-related microorganisms in the paddy and purple soils

**Figure 3:**
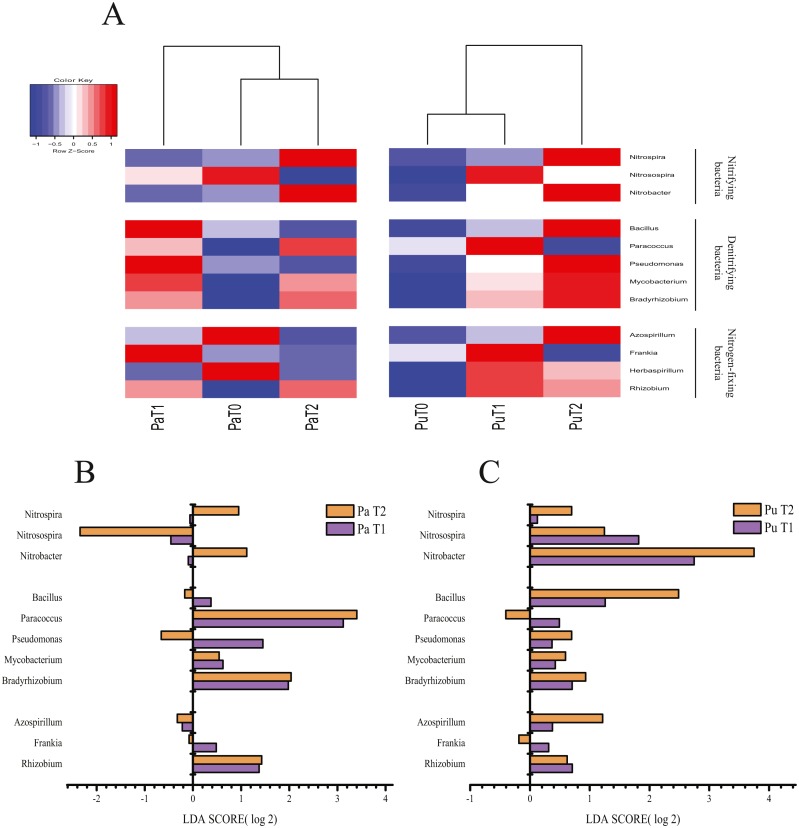
Effect of straw biochar application on N-related microorganisms in the paddy and purple soils. (A) Heatmap of N-related microorganisms in the paddy and purple soils; (B) fold change of N-related microorganisms in the paddy soil; (C) fold change of N-related microorganisms in the purple soil.

In this study, 16sDNA sequencing was used to analyze the changes in the abundance of microorganisms related to nitrogen metabolism in the purple and paddy soil samples. Among all the samples, we detected 2,336,413 tags, ranging from 47,553 to 90,581 tags per sample. Operational taxonomic unit (OTU) clustering resulted in 209,276 OTUs (Table S3). Biochar application altered microbial community composition and increased bacterial diversity in the soil. Venn diagrams based on the OTUs revealed that 2252 OTUs were shared between the control and treatments (T0, T1, and T2) in the paddy soil (T0: 8566 OTUs; T1: 8996 OTUs; T2: 8627 OTUs; Fig. 2A). In the purple soil, 2384 were shared between the control and treatments (T0: 9622 OTUs; T1: 10 056; T2: 9922 OTUs; Fig. 2B). Biochar application caused the alpha-diversity of the microbial community to increase significantly in the purple soil (LSD test, *P* = 0.0026) (Fig. 3E), but not in the paddy soil. Biochar application increased the beta-diversity, making the microbial communities in the paddy and purple soils more distinct. The principle coordinate analysis based on weighted UniFrac distances revealed significant separation of the three treatments along the primary principal coordinate in both soil types (Figs. 3C and 3D). The increase in diversity following biochar application occurred because the application of biochar to the soil improved the amount of nutrients, including soil organic matter, which are used by soil microorganisms for growth and reproduction (Luo & Sun, 1994; Pietikäinen, Kiikkilä & Fritze, 2000). Additionally, biochar in the soil can change the soil environment and microbial habitat, affecting the microbial community (Jones et al., 2012). These results correspond with those of previous studies (Rondon et al., 2007; Wang et al., 2017).

Analysis of OTU annotations in each sample revealed that the abundance of microbes involved in N cycling changed after biochar application in both soil types (Fig. 3A), but the effect differed between the purple and paddy soils (Figs. 3B and 3C). Biochar application possibly improved the abundance of nitrifying and denitrifying bacteria by improving the soil properties (Abujabhah et al., 2018). In the present study, the application of biochar for two consecutive years increased nitrifying bacterial abundance more significantly than the application of biochar for one year in the paddy soil. Therefore, the effect of biochar on N-poor paddy soil may accumulate over the years. The abundance of *Nitrosospira* was significantly reduced in the paddy soil, but it was increased in the purple soil. This could be explained by the differences between the in the initial properties and properties following biochar application. Denitrification, the process by which microorganisms use N oxides as electron acceptors (Tang et al., 2016), is of crucial significance in the natural N cycle. Our finding that biochar significantly improved the abundance of denitrifying bacteria in both paddy and purple soils is consistent with that of a previous study (Duan et al., 2018). Several studies have shown that biochar application increases the abundance of N-fixing bacteria (Lehmann et al., 2008; Singh et al., 2010). This effect is more noticeable in lighter-textured soils (Chen et al., 2017), such as the purple soil that we studied; this is possibly because biochar application increases the C/N ratio and nutrient availability, especially of Bo and Mo (Rondon et al., 2007), elements that are important for N-fixing bacteria. In addition, biochar application can improve the physical properties of the soil, and thereby, improve the abundance and increase the activity of free-living N-fixing bacteria such as *Azospirillum* (Abujabhah et al., 2018).

**Figure 4:**
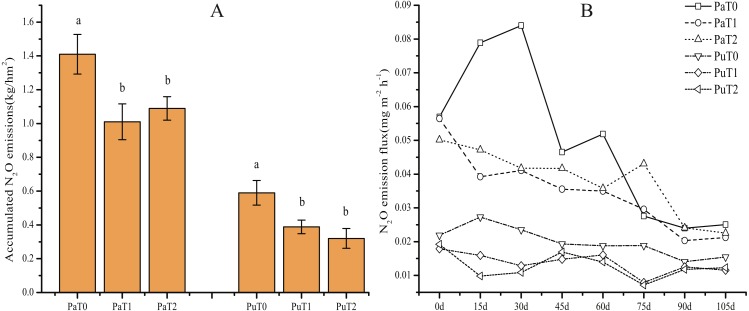
Effect of straw biochar application on N_2_O emission in the paddy and purple soils. The letters above the bars identify groups of treatments between which the values differ significantly at the 0.05 significance level (LSD test).

### Effect of straw biochar on N_2_O emission in the paddy and purple soils

It has been shown that straw biochar is more effective than other forms of biochar (e.g., wood and husk) in reducing greenhouse gas emission (He et al., 2017; Song et al., 2016). Our finding that straw biochar application suppressed N_2_O emission (Figs. 4A and 4B) is consistent with their findings. As shown in Fig. 4A, the application of straw biochar significantly suppressed N_2_O emission in the paddy and purple soils. After 1 year of straw biochar application, N_2_O emission from paddy and purple soils was reduced by 28.37% (*P* < 0.00048) and 30.79% (*P* < 0.00082), respectively, relative to that of the control treatment; the reduction after 2 years of biochar application was 22.70% (*P* < 0.00079) and 52.54% (*P* < 0.00011), respectively. During the growth period of tobacco plants, the peak N_2_O emission period was from transplant to 60 days after transplanting (Fig. 4B). Among them, the difference between 1 year of application and two years of continuous application was not obvious, but the application of biochar can reduce the release of soil N_2_O compared with that of the treatment without biochar. Biochar can serve as an electron donor during denitrification, and can thereby, create anaerobic micro-field conditions that are favorable for denitrification (Anderson et al., 2011). This fact explains the reduction in N_2_O emission that we observed.

Biochar application improved the abundance of denitrifying bacteria such as *Mycobacterium* and *Bradyrhizobium*, which have the *narG* and *norZ* functional genes, respectively (Figs. 3C and 3D). Our finding that *Mycobacterium* and *Bradyrhizobium* increased in abundance following biochar application is consistent with earlier study results (Anderson et al., 2011; Yanai, Toyota & Okazaki, 2007). These bacteria can restore nitrate levels in the soil and catalyze the conversion of N_2_O into N_2_ (Anderson et al., 2011), which contributes to enhanced generation of N_2_. However, the change in bacterial abundance did not differ between 1 and 2 years after biochar application, in either paddy or purple soil, suggesting that the release of soil N_2_O is affected by the timing of biochar application; biochar that is applied during the growing season affected soil N_2_O, whereas biochar applied in the previous growing season did not. The abundance of *Mycobacterium* and *Bradyrhizobium* did not differ between 1 and 2 years after biochar applications, in either soil type. This suggests that biochar has limited effect on the abundance of bacteria involved in N cycling.

### Effect of straw biochar on the tobacco yield

**Figure 5:**
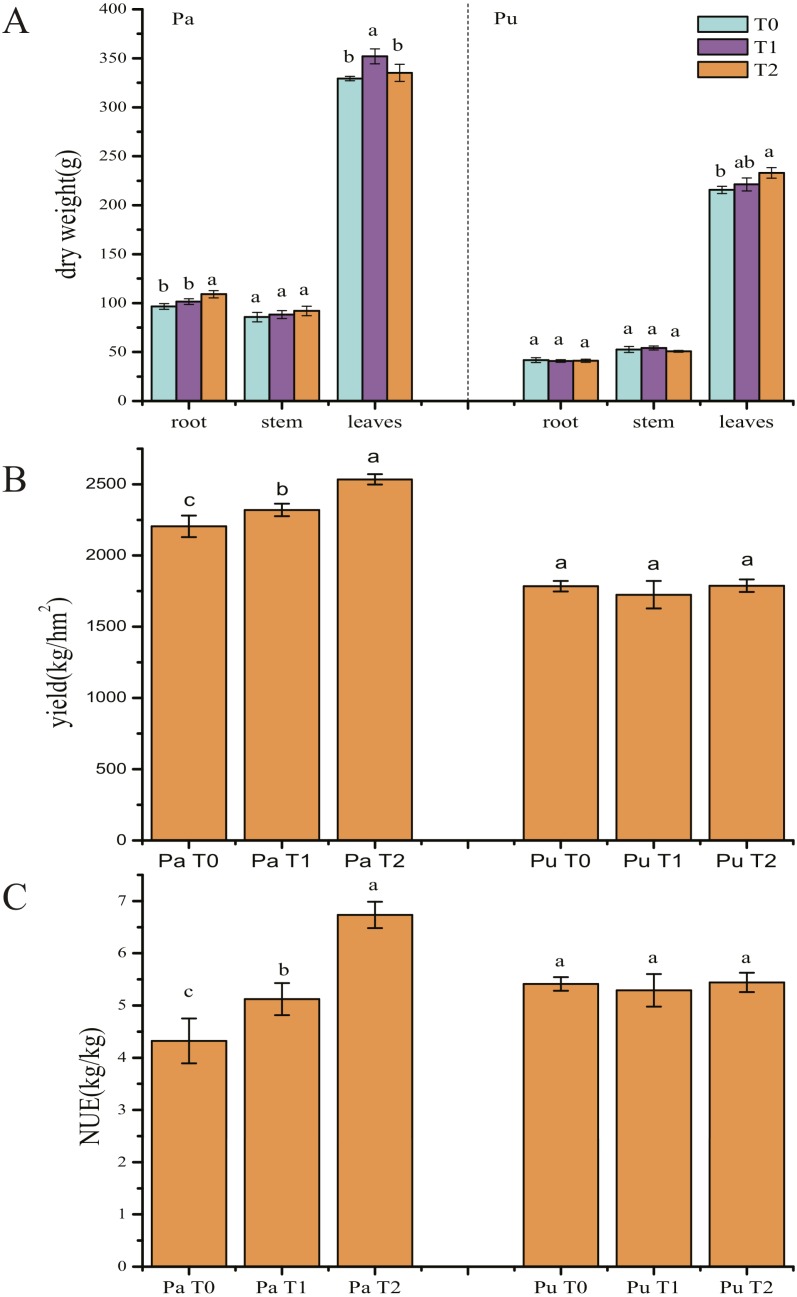
Effect of straw biochar application on tobacco plants in the paddy and purple soils. (A) Dry weight; (B) yield; (C) nitrogen use efficiency (NUE).

Biochar application significantly affected tobacco plants in paddy soil, but had negligible effect in the purple soil. In the paddy soil, but not in the purple soil, biochar application improved the dry weight of tobacco root (5.21%, *P* = 0.13; 12.94%, *P* = 0.017;) leaf (6.89%, *P* = 0.0077; 1.76%, *P* = 0.34) after 1 and 2 years of biochar application, respectively, relative to that of the control treatment. Furthermore, biochar application significantly increased tobacco yield, with a considerably greater effect after 2 years than after 1 year, which was 5.19% (*P* = 0.037) for 1 year and 14.92% (*P* = 0.0012) for 2 years (Figs. 5A, 5B, and SI 5). The same trend occurred in terms of NUE, which was improved by 18.49% (*P* = 0.0093) for 1 year and 34.34% (*P* = 0.00016) for 2 years. Our finding that biochar increased tobacco yield indicates that ongoing application of biochar promotes tobacco growth (Chen & Du, 2015; Xiefeng et al., 2015), by improving the N pool and raising soil pH, and thereby increasing the uptake of soil nutrients by tobacco. The fact that biochar did not increase yield in purple soil may be because purple soil is alkaline, and biochar raises the soil pH further. Higher pH is not conducive for the growth of tobacco roots, thus hindering nutrient uptake. This is consistent with the results of previous studies (Chen & Du, 2015; Jiangzhou et al., 2017).

## Conclusions

We found that biochar application to the purple and paddy soils promoted the process of soil N transformation. In particular, it reduced the loss of N fertilizer through leaching of ammonium- and nitrate-derived N especially in the paddy soil, thereby promoting the growth of tobacco plants. In addition, it improved the abundance of bacteria involved in the N cycle, particularly denitrifying bacteria, which help decrease the emission of N_2_O by conversion of N_2_O into N_2_. Therefore, biochar application is beneficial in agriculture because it reduces the amount of N fertilizer required during cultivation. Biochar application alleviates problems related to N pollution that can arise from high rates of fertilizer application and low rates of fertilizer utilization in southern areas of China. In the future, we suggest continuing trinity research of long-term biochar application, soil, and plant.

##  Supplemental Information

10.7717/peerj.7576/supp-1 Table S1 Best tobacco yield by previous test

10.7717/peerj.7576/supp-2 Table S2 Field work information

10.7717/peerj.7576/supp-3 Table S3 The properties of straw biochar

10.7717/peerj.7576/supp-4 Table S4 OTUs and Tag numbers for different soil samples

10.7717/peerj.7576/supp-5 Figure S1 Tobacco yield for no N applied in paddy and purple soil

## Additional Information and Declarations

### Data Availability

The following information was supplied regarding data availability: Data is available at NCBI, accession number: PRJNA512246.
