# Supplementary material for: Biochar application significantly affects the N pool and microbial community structure in purple and paddy soils
Source: PeerJ. 2019 Sep 13;7:e7576. doi: 10.7717/peerj.7576 (PMC6746220; doi:10.7717/peerj.7576)
Supplement: Table S1 [file peerj-07-7576-s001.docx]

**Table SI1** The effect of biochar amount application on yield, output value and average price of tobacco plants.

| treatment | | Yield  (kg/hm^2^) | output value  (yuan/hm^2^) | Average price  (yuan/kg) |
| --- | --- | --- | --- | --- |
| paddy soil | T1 | 2175.00d | 40392.60b | 18.57b |
|  | T2 | 2299.80c | 40288.80b | 17.52c |
|  | T3 | 2605.05a | 49536.00a | 19.01ab |
|  | T4 | 2441.25b | 47685.00a | 19.53a |
| purple soil | T1 | 1997.55b | 37670.10b | 18.86b |
|  | T2 | 1997.70b | 38250.30b | 19.15ab |
|  | T3 | 2165.55a | 43021.05a | 19.87a |
|  | T4 | 2113.65a | 41069.55a | 19.43a |

* different letters indicate significant difference (LSD, p < 0.05);

* T1, T2, T3 and T4 represent 5.4t/ha, 6.3t/ha, 7.2t/ha and 8.1t/ha biochar application.
