# Supplementary material for: Biochar application significantly affects the N pool and microbial community structure in purple and paddy soils
Source: PeerJ. 2019 Sep 13;7:e7576. doi: 10.7717/peerj.7576 (PMC6746220; doi:10.7717/peerj.7576)
Supplement: Table S2 [file peerj-07-7576-s002.docx]

**Table SI2** The information of the experimental design

| treatment | | tobacco  species | transplanting date  (mm/dd/yy) | cultivation and earthing up  (mm/dd/yy) | last day for harvest | growth period |
| --- | --- | --- | --- | --- | --- | --- |
| paddy soil | T0 | Yunyan 87 | 03/01/2017 | 03/20/2017  04/15/2017 | 07/08/2017 | 130 |
|  | T1 | Yunyan 87 | 03/01/2017 | 03/20/2017  04/15/2017 | 07/08/2017 | 130 |
|  | T2 | Yunyan 87 | 03/05/2016 | 03/25/2016  04/20/2016 | 07/14/2016 | 132 |
|  |  | Yunyan 88 | 03/01/2017 | 03/20/2017  04/15/2017 | 07/08/2017 | 130 |
| purple soil | T0 | Yunyan 89 | 03/02/2017 | 03/20/2017  04/15/2017 | 07/06/2017 | 128 |
|  | T1 | Yunyan 90 | 03/02/2017 | 03/20/2017  04/15/2017 | 07/06/2017 | 128 |
|  | T2 | Yunyan 91 | 03/06/2016 | 03/25/2016  04/20/2016 | 07/10/2016 | 128 |
|  |  | Yunyan 92 | 03/02/2017 | 03/20/2017  04/15/2017 | 07/06/2017 | 128 |
