# Supplementary material for: Biochar application significantly affects the N pool and microbial community structure in purple and paddy soils
Source: PeerJ. 2019 Sep 13;7:e7576. doi: 10.7717/peerj.7576 (PMC6746220; doi:10.7717/peerj.7576)
Supplement: Table S3 [file peerj-07-7576-s003.docx]

**Table S3** The properties of straw biochar

| Variable | Biochar |
| --- | --- |
| Total C (%) | 52.8 |
| Total N (%) | 0.41 |
| pH | 9.36 |
| Ash (%) | 11.2 |
| Specific surface area（m^2^ g^-1^） | 16.72 |
| Nitrate-N (mg/kg) | 0.38 |
| Aluminum (%) | 0.17 |
| Arsenic (mg/kg) | <4 |
| Boron (mg/kg) | 13 |
| Calcium (%) | 5.6 |
| Cadmium (mg/kg) | <0.15 |
| Cobalt (mg/kg) | 4.3 |
| Chromium (mg/kg) | 7.9 |
| Copper (mg/kg) | 26 |
| Iron (%) | 0.5 |
| Potassium (%) | 0.41 |
| Magnesium (%) | 1.22 |
| Manganese (mg/kg) | 1893 |
| Molybdenum (mg/kg) | 1.8 |
| Sodium (%) | 0.16 |
| Nickel (mg/kg) | 5.8 |
| Phosphorus (%) | 2.3 |
| Lead (mg/kg) | 3.7 |
| Sulfur (%) | 0.39 |
| Selenium (mg/kg) | <3 |
| Zinc (mg/kg) | 117 |
