# Supplementary figures and images for: Biochar application significantly affects the N pool and microbial community structure in purple and paddy soils
Source: PeerJ. 2019 Sep 13;7:e7576. doi: 10.7717/peerj.7576 (PMC6746220; doi:10.7717/peerj.7576)

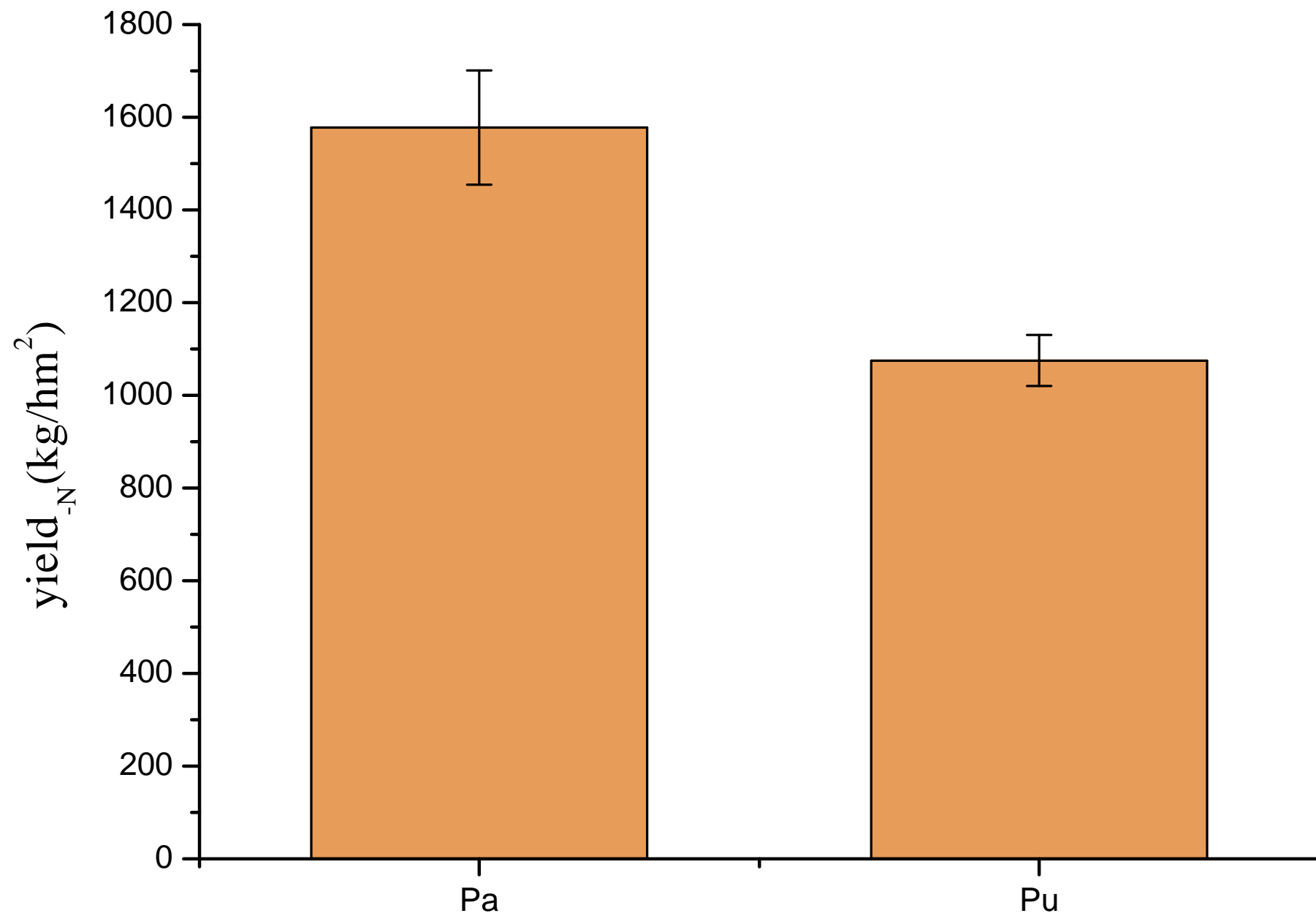

Supplement: Figure S1 [file peerj-07-7576-s005.pdf]
